# Improving teaching on the basis of student evaluation: Integrative teaching consultation

**DOI:** 10.3205/zma000944

**Published:** 2015-02-11

**Authors:** Gerald Wibbecke, Janine Kahmann, Tanja Pignotti, Leander Altenberger, Martina Kadmon

**Affiliations:** 1 Medizinische Fakultät Heidelberg, Zentrale Evaluation und Integrative Lehrberatung, Heidelberg, Germany; 2 Universität Heidelberg, Zentrale Studienberatung/Career Service, Heidelberg, Germany; 3 Carl-von-Ossietzky Universität Oldenburg, Campus Wechloy, Gebäude W16a, Oldenburg, Germany

**Keywords:** Teaching consultation, student evaluation, faculty development

## Abstract

**Objective: **Due to the development of medical education in the past decade the role of teachers has changed and requires higher didactic competence. Student evaluation of teaching alone does not lead to considerable improvement of teaching quality. We present the concept of "Integrative Teaching Consultation", which comprises both the teacher’s reflection and own objectives to improve their teaching as well as data from students ratings.

**Methods: **Teachers in collaboration with a teaching consultant reflect on their teaching ability and set themselves improvement goals. Then the consultant himself observes a teaching session and subsequently analyses the respective student evaluation in order to give meaningful feedback to the teacher.

**Results: **The combination of student feedback with professional consultation elements can initiate and maintain improvements in teaching.

**Conclusion: **Teaching consultation complements existing faculty development programs and increases the benefit of student evaluations.

## Zusammenfassung

**Zielsetzung:** Die Weiterentwicklung der medizinischen Lehre hat die Erwartungen an die Lehrkompetenz der Lehrenden erhöht. Da der flächendeckende Einsatz studentischer Lehrveranstaltungsevaluationen allein weder zu einer Verbesserung der Lehre noch zu einer Weiterentwicklung der persönlichen Lehrkompetenz führt, haben wir das Konzept der Integrativen Lehrberatung entwickelt.

**Methodik: **Lehrende erarbeiten mit professionellen Lehrberatern das Verbesserungspotential ihrer Lehre und setzen sich selbst didaktische Ziele. Anschließend hospitieren Lehrberater die Lehrveranstaltungen, werten die studentische Lehrveranstaltungsevaluation inhaltsanalytisch aus und geben den Dozenten ein Expertenfeedback.

**Ergebnisse: **Die Integrative Lehrberatung kann durch die Kombination von professioneller Beratung und eigenen Zielsetzungen nachhaltige Verbesserungen der Lehre in Gang setzen.

**Schlussfolgerung: **Das Konzept der Integrativen Lehrberatung unterstützt bestehende Fakultätsentwicklungsprogramme und bietet eine effiziente Möglichkeit, studentisches Feedback in das Qualitätsmanagement der Lehre zu integrieren.

## Introduction: how education can be improved by evaluation

Parallel to the paradigm *shift from teaching to learning* [1] the role expectations towards teachers change [2], [3]. They are far more required to support self-directed learning among students. Competency frameworks for teachers include the ability to flexibly select suitable didactic strategies towards this end [4]. At the same time they assume that teachers acquire a student-centered approach to teaching only through experience [5], [6]. This includes the ability to reflect on the impact of their teaching from a student perspective. To become a reflective practitioner requires continuous professional development. Teachers are often not prepared for this challenging task [7], even experienced and committed medical teachers see a need for further qualification for themselves [8]. 

As an essential part of faculty development programs, workshops and seminars have been designed to improve teacher effectiveness. While the literature describes successful programs, their long-term effect and the sustainability remain unclear [9]. The transfer of theory into practice and the anticipation and handling of obstacles remain major challenges. Communication between teachers and students about teaching and students’ needs is an important prerequisite for successful teaching and learning [10]. Lecturers usually receive systematic feedback by questionnaire-based surveys of their students [11]. Student evaluations of teaching (SET) are often used to assess teaching quality at universities [12]. However, neither numerical evaluation data nor additional free-text-comments of students provide a sound enough basis for improvement. Our experience with free text comments shows that students can name weaknesses of teaching sessions, but they are not able to stimulate new ways of teaching. These limitations of SETs are confirmed by empirical data. SET feedback does not lead to better evaluation results [13], [14] or to a perceived change in teaching quality [15]. In order to support curricular development and change as well as teachers’ ability to improve their teaching habits, further interventions are necessary.

## Project description

The "Integrative Teaching Consultation" (ITC) is a team of pedagogues and psychologists headed by a medical doctor. The aim of the ITC is to combine SETs with counseling and professional feedback in order to enhance the ability of teachers to translate the SET feedback into concrete measures of teaching behavior. Evidence on teaching and learning and principles of effective consultative feedback are used during consultation. This includes active involvement of teachers in the counseling process, the use of teacher self-ratings and feedback from teaching observations [16]. Studies have shown over 30 years that consultative feedback interventions have an impact on teaching effectiveness [17], [18], [19], but are rarely implemented at universities. 

**Figure 1:**
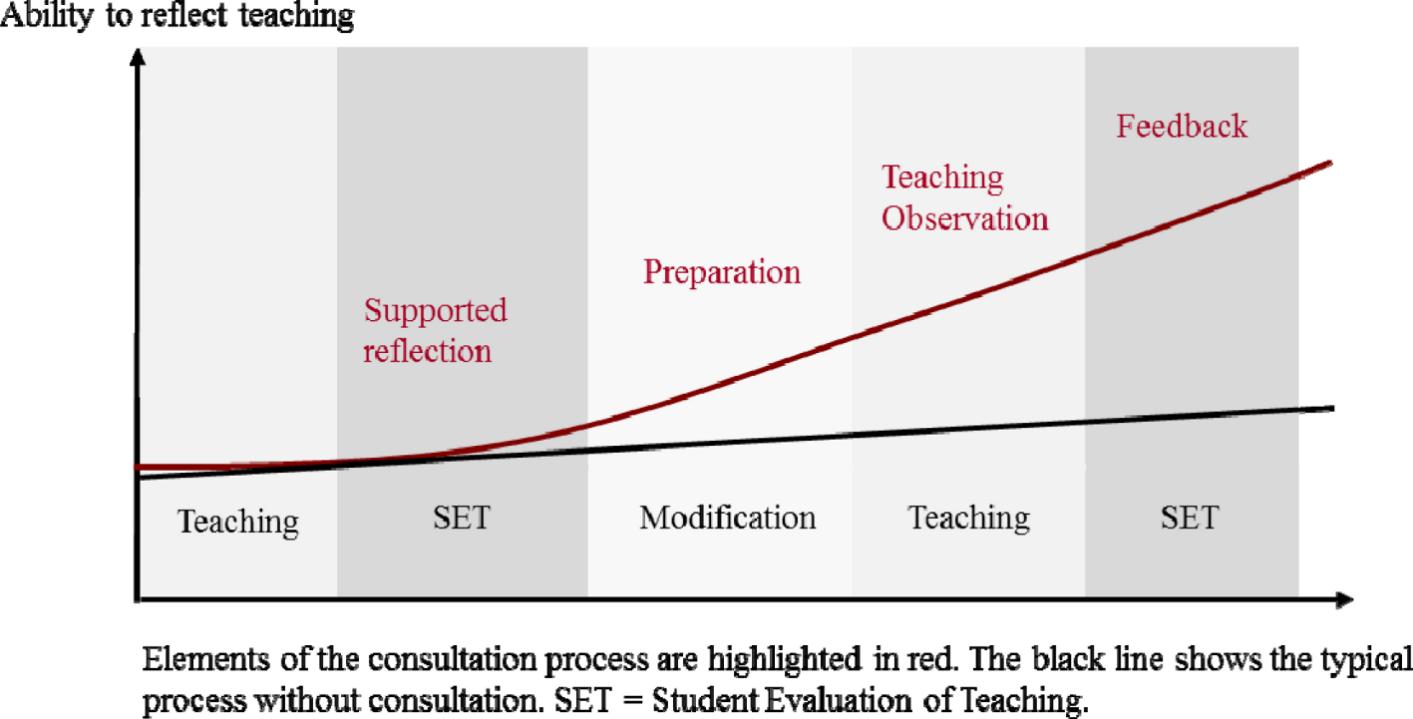
Process of the ITC

At the Medical Faculty of Heidelberg all departments receive additional feedback from the ITC on the basis of SETs. Successful lecturers receive positive feedback on their key factors of success. Lecturers with less positive evaluation results receive didactical advice to improve their teaching, as well as an offer for teaching consultation. The goal of the ITC is to support the teachers individually in order to improve their teaching and to strengthen their ability of self-assessment (see figure 1 (Fig. 1)). During the first appointment, teachers report which challenges they experience as the most important ones and on which the focus during the subsequent classroom observation should be put (supported reflection). Thus, they set improvement goals for themselves. Furthermore, SETs are analyzed together with the consultant. The second step (preparation) includes planning of the teaching session ahead and, optionally, training measures. The focus in this phase may be revision of teaching material or planning specific didactic activities. Finally, the teaching observation with the previously defined observation aspects takes place (teaching observation). The results are then being discussed in a personal encounter in order to detect and agree on possibilities for further development. During this feedback situation the results of the SETs are taken into consideration (feedback and SET). 

### Lecturers as Reflecting Practitioners

If the consultation is successful, the lecturers improve on their ability to reflect their own teaching and may, hence, develop measures to advance their teaching independently. This way, student-centered teaching may be adopted. Ideally, the effectiveness of consultation should show in subsequent SETs. The approach of the ITC will be further illustrated in the following example.

## Outcome. An example from practice

SETs revealed need for improvement in a scientific, preclinical subject. Two professors shared a series of lectures (each of them taught six), which were complemented by practical training sessions and tutorials. One of the lecturers sought the support of the ITC in order to aim to adapt and improve his lectures and tutorials. On the basis of his reflections, the feedback and the SETs, he implemented concrete changes in his lectures and tutorials: learning objectives were clearly defined and communicated to the students in order to enhance the transparency of content and success criteria. Application exercises related to practice were integrated into the seminars to stress the importance of certain learning objectives and to uncover possible queries of the learners during the lecture. At the same time the content of the lessons was reduced. Tutorials were adapted with the help of assistant tutors who had received good evaluations in the past. The entire counseling process including teaching observation, pre- and post-processing extended over a period of 18 months.

To analyze the effectiveness of the consultation process, the free texts of the students relating to the didactic lecture and the tutorials were analyzed using the method of Mayring [20]. The texts were abstracted step-by-step and summarized. In order to compare SETs at different time points, free text statements were assigned to three different categories: Praise (positive comments on teaching), criticism (negative comments on teaching) and recommendationss (ideas for improvement). The sum of the statements in each category was related to the total sum of statements. The data from three different SETs in three different semesters were included in the analysis. The first SET (t1) took place before the consultation, the second one (t2) six months after the beginning of the consultation process and the last one (t3) two years after the first evaluation. 

The results (see table 1 (Tab. 1)) indicate a clear shift in the free text statements. The number of positive statements (praise) increased both in the lecture of the teacher who had engaged in the counseling (from 1.1% on t1 to 10.9% on t3) and in the respective tutorials (from 10% on t1 to 20.3% on t3). The amount of criticisms and recommendations decreased. The teaching staff who had participated in the consultation process reached better subsequent evaluation results. Quality of explanations (n=59) and learning atmosphere (n=58) were the most frequently mentioned positive aspects. Recent studies on teaching consultation suggest that teaching competency may well be estimated on the basis of SETs [13], [14]. The comparability of the evaluation results is somewhat limited because they come from different student cohorts at the three evaluation time points. However, since admissions criteria were constant, we assume homogeneity of the student cohorts and their judgment. Whether the higher response rate at t3 causes a distortion of results remains an open question.

## Conclusion

If SETs alone do not lead to considerable improvement of teaching quality, it should not be the centerpiece of quality assurance. Combined with feedback interventions, the effectiveness of SETs can be improved. Our findings suggest that consultation on the basis of student ratings is an effective method for enhancing the teaching effectiveness of university teachers.

This is based on the prerequisite that a long-term support of lecturers and disciplines is possible. The close connection between faculty consultant, faculty development and the SETs seems to be important. The concept of ITC may also be applied to other faculties. For the improvement of teaching quality, a teaching consultation should be integrated into the quality management of medical universities. Future research should attempt to clarify, what changes can be expected and which factor of consultation is notably relevant for lecturers and the curriculum. 

## Competing interests

The authors declare that they have no competing interests.

## Figures and Tables

**Table 1:**
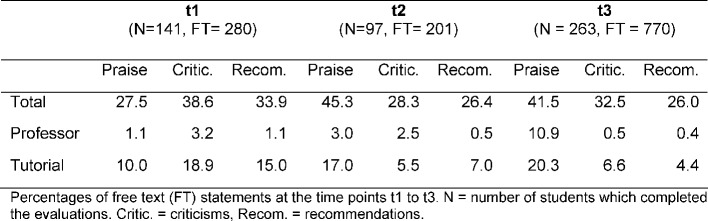
Analysis of the free text comments before and after the consultation.
